# DEVELOPMENT OF THE SWALLOWING ACTIVITY CLASSIFICATION MODEL FOR OLD ADULTS BASED ON ACOUSTIC ANALYSIS

**DOI:** 10.1093/geroni/igae098.2991

**Published:** 2024-12-31

**Authors:** Dan Li, Yaqing Zhang, Junhui Wu, Wei Luo, Tao Liu, Benshuai Lyu, Shaomei Shang

**Affiliations:** Peking University, Beijing, Beijing, China (People’s Republic); Peking University, Beijing, Beijing, China (People’s Republic); Peking University Health Science Center, Beijing, Beijing, China (People’s Republic); Peking University Health Science Center, Beijing, Beijing, China (People’s Republic); Peking University Health Science Center, Beijing, Beijing, China (People’s Republic); Peking University, Beijing, Beijing, China (People’s Republic); Peking University Health Science Center, Beijing, Beijing, China (People’s Republic)

## Abstract

Dysphagia poses a significant health threat for old people worldwide, often resulting in severe complications like aspiration, choking, and aspiration pneumonia, thereby increasing hospital readmission rates and mortality. Swallowing function assessment serves as an effective means to reduce the occurrence of complications and improve patients’ survival rate. However, current assessment tools have pronounced limitations. Acoustic diagnosis, as a non-invasive method, has shown promising effectiveness in dysphagia screening and aspiration detecting, rendering it a key technology for addressing this issue. This study aims to develop a swallowing activity classifier based on acoustic recording and analysis using machine learning algorithms, which constitutes an efficient tool for non-invasive, remote, and continuous assessment of swallowing activities in old adults. The study recorded and annotated swallowing audio data from 61 old individuals, resulting in 120 acoustic samples. Signal features in time, frequency, time-frequency, and statistical domains were analyzed and extracted as inputs. Multiple models, including the Multi-Layer Perceptron (MLP), Convolutional Neural Network (CNN), and Convolutional Recurrent Neural Network (CRNN), were used for classification. The MLP, CNN, and CRNN models achieved accuracy values of 74%, 68%, and 54%, respectively, on the validation set. Results indicate that swallowing, coughing, throat clearing, choking, wet breathing, and speech are characterized by distinct acoustic patterns, which can be effectively classified and identified using machine learning methods. These findings hold promise for the research and practical application of acoustic and machine learning-based swallowing activity classification models, facilitating continuous monitoring in old people, thereby providing guidance for clinical treatment and care.

